# Tirzepatide, a New Era of Dual-Targeted Treatment for Diabetes and Obesity: A Mini-Review

**DOI:** 10.3390/molecules27134315

**Published:** 2022-07-05

**Authors:** Vivek P. Chavda, Jinal Ajabiya, Divya Teli, Joanna Bojarska, Vasso Apostolopoulos

**Affiliations:** 1 Department of Pharmaceutics and Pharmaceutical Technology, LM College of Pharmacy, Ahmedabad 380009, India; 2 Department of Pharmaceutics Analysis and Quality Assurance, LM College of Pharmacy, Ahmedabad 380009, India; 98jinal@gmail.com; 3 Department of Pharmaceutical Chemistry, LM College of Pharmacy, Ahmedabad 380009, India; divya.teli@lmcp.ac.in; 4 Institute of General and Ecological Chemistry, Faculty of Chemistry, Lodz University of Technology, 116 Żeromskiego Street, 90-924 Lodz, Poland; 5 Institute for Health and Sport, Victoria University, Melbourne, VIC 3030, Australia; 6 Australian Institute for Musculoskeletal Science (AIMSS), Immunology Program, Melbourne, VIC 3030, Australia

**Keywords:** tirzepatide, twincretin, short peptide, incretins, diabetes, obesity

## Abstract

The prevalence of obesity and diabetes is an increasing global problem, especially in developed countries, and is referred to as the twin epidemics. As such, advanced treatment approaches are needed. Tirzepatide, known as a ‘twincretin’, is a ‘first-in-class’ and the only dual glucagon-like peptide-1 (GLP-1) and glucose-dependent insulinotropic peptide (GIP) receptor agonist, that can significantly reduce glycemic levels and improve insulin sensitivity, as well as reducing body weight by more than 20% and improving lipid metabolism. This novel anti-diabetic drug is a synthetic peptide analog of the human GIP hormone with a C_20_ fatty-diacid portion attached which, via acylation technology, can bind to albumin in order to provide a dose of the drug, by means of subcutaneous injection, once a week, which is appropriate to its a half-life of about five days. Tirzepatide, developed by Eli Lilly, was approved, under the brand name Mounjaro, by the United States Food and Drug Administration in May 2022. This started the ‘twincretin’ era of enormously important and appealing dual therapeutic options for diabetes and obesity, as well as advanced management of closely related cardiometabolic settings, which constitute the leading cause of morbidity, disability, and mortality worldwide. Herein, we present the key characteristics of tirzepatide in terms of synthesis, structure, and activity, bearing in mind its advantages and shortcomings. Furthermore, we briefly trace the evolution of this kind of medical agent and discuss the development of clinical studies.

## 1. Introduction

Diabetes and obesity are chronic diseases leading to substantial morbidity and high mortality worldwide, especially in developed countries. They are considered the twin epidemics of the 21st century. Neither disorder is a simple problem; rather, both are complex health issues combining genetic, epigenetic, and lifestyle factors, including socioeconomic and environmental impacts [1,2,3].

Obesity is defined by the World Health Organization (WHO) as an “abnormal or excessive fat accumulation that may impair health” [4,5]. It is defined by body mass index (BMI), weight in kilograms divided by the square of height in meters, in adults over 30 kg /m^2^ [6]. It should be highlighted that obesity is related to an increased risk of other serious conditions and diseases such as diabetes, hypertension, cardiovascular disease, cancer, asthma, hypercholesterolemia, and so on [7]. To date, the fight against obesity has been one of the greatest challenges. Treatment has been based only on a well-balanced diet and regular physical activity, so far.

Type 2 diabetes (T2D) is an incurable condition that influences glucose regulation in the body. Cases are increasing daily at a menacing rate. The rise in the number of T2D cases over a period of 34 years (1980–2014) was four-fold [8], with a 5% increase in premature mortality from T2D from 2000 to 2016. Due to 1.5 million estimated deaths being directly caused by T2D, it was termed the ninth-biggest mode of mortality in 2019 [9]. A whopping 537 million adults across the globe were suffering from T2D in the year 2021, and this number is estimated to rise to 783 million by the year 2045 (with a surge of 12.2%) [10]. These statistics reveal that currently, 1 in 10 adults are suffering from T2D, and this number is increasing at pandemic rates.

Beta cells that produce insulin, present in the pancreas, play a significant role in the origination and development of T2D by controlling the endocrine system for glucose metabolism and glycemia. In T2D, these cells become inoperative in order to compensate for insulin resistance, resulting in an insulin-deficient condition called hyperglycemia. Hyperglycemia can result in glucose toxicity that further deteriorates the function of beta cells of the pancreas, giving rise to a deficiency of insulin in the body [11].

Treatment of T2D includes lifestyle changes such as diet, exercise, and nutraceuticals, as well as administration of medications such as metformin. Various synthetic moieties and herbal preparations have been developed to support the functioning of beta cells and suppress harmful inflammatory responses [12]. The majority of these agents have unwanted effects pertaining to the route of administration and various other factors. The common side effects observed with the sulfonyl urea class of drugs include syncope, dizziness, nervousness, anxiety, depression, and diarrhea. The adverse effect most associated with metformin is gastrointestinal upset, and with repaglinide this is hypoglycemia [13,14]. In such cases, only a small amount of medication reaches the cells. The therapeutic effect of such kinds of drugs can be rapidly improved by targeting their delivery to the pancreatic islets or by lowering the dose consumed, as well as by decreasing the side effects of systematically administered agents [15].

Conventionally, two sorts of therapeutic agents have been used for the treatment of T2D: insulin or its analogs, such as insulin lispro, insulin aspart, and oral agents like glipizide, glimepride, metformin, acarbose, pioglitazone, saxagliptin [16]. In addition to these agents, a short-acting insulin that can be inhaled just before nutrient intake—Exubera^®^—was approved by the United States Food and Drug Administration (US FDA) in January 2006 for therapeutic use [17].

**Figure 1:**
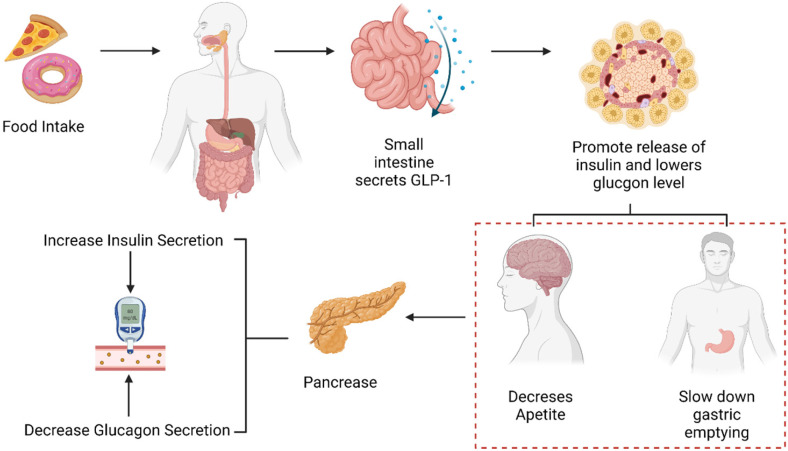
Role of GLP-1 in glucose metabolism.

Furthermore, products generated in the human body that stimulate beta cells to release insulin, termed as ‘Incretins’, also known as ‘Incretin hormones’, were discovered in the early 1970s. These products are secreted in the intestine, affecting the functioning of beta cells (Figure 1). The most commonly known incretins include glucagon-like peptide-1 (GLP-1) and glucose-dependent insulinotropic polypeptide (GIP) [18,19].

The adverse drug reaction profile of tirzepatide is comparable to GLP-1 agonists, as it is a dual GIP/GLP-1 agonist. The most common side effects associated with tirzepatide are related to gastrointestinal tract like nausea, vomiting and diarrhea [20,21]. The incidence of hypoglycemia is low, based on phase 2 trials [20].

The focus of this mini-review is to present a brief overview of the synthetic short peptide tirzepatide as the first dual GLP-1 and GIP receptor agonist as a promising therapeutic agent for the treatment of either diabetes or obesity and to highlight its superiority to other similar agents. Notably, modified short peptides have improved bio-functions compared with those in the original analogs, and are of great promise in modern biomedicine thanks to recent advances in science and biotechnology [22]. The most up-to-date pharmacologic characterization of tirzepatide and its clinical evolution are discussed.

## 2. Methodology

For the preparation of this review, we carried out a literature search using SciFinder^®^, PubMed, and Scopus. The key words ‘tirzepatide and Diabetes’, ‘tirzepatide and Obesity’, ‘novel antidiabetics’ were used to search the related articles. In total, 90 articles were found containing the two concepts ‘tirzepatide’ and ‘Diabetes/Obesity’ closely associated with one another. From these, we included those articles that employed synthetic methods, in vivo study, or discussion of tirzepatide clinical trials. No conference abstracts or book chapters were included, and only papers written in English language were included.

## 3. Insulinotropic Peptides

The effect caused by these insulinotropic peptides is called the ‘incretin effect’. This effect is characterized by elevation of glucose secretory response by oral glucose administration in comparison to intravenous administration even though they have similar plasma concentrations. The name is given because it is believed to occur due to nutrients triggering the release of incretin hormones and also acting upon pancreatic beta cells in an insulinotropic manner (Figure 1) [23].

GLP-1 and GIP are peptide hormones that are secreted with the benefit of utilizing cells from intestines called enteroendocrine cells in reaction to the consumption of nutrients, and they have an important function in postprandial metabolism. With the identification of glucose equilibrium, their most favorable condition, the incretin effect, begins to improve the glucose-stimulated release of insulin from the pancreas. GIP is considered to be the essential incretin hormone accounting for this effect, although when administered together, they exhibit a synergetic effect [24].

The dipeptidylpeptidase-4 (DPP-4) inhibitors act by obstructing the quick DPP-4-mediated degradation of endogenous GLP-1 and GIP, enhancing their effectiveness. Meanwhile, the GLP-1 receptor agonists (RA), which have the advantage of an alteration in structure, create resistance against the degradation of DPP-4 by intensifying the onset of the GLP-1 receptor [25]. GLP-1 RAs also assist in the reduction of glucagon secretion of the pancreas through alpha cells, reducing gastric emptying time and reducing appetite and intake of nutrients, thus inducing weight loss in a manner different from glucose-dependent stimulation. In contrast to externally administered GLP-1, which partly restores incretin action in T2D patients, GIP infusion does not bring out a significant insulin secretory response, even at pharmacological concentrations [24]. For treatment of T2D, GIP-selective RAs have not been widely considered, largely based on these findings.

Noteworthy progress has been made in this therapeutic class since the Exenatide injection, produced by Amylin Pharmaceuticals, Inc. and Eli Lilly and Company, marketed under the name Byetta^®^, which is a GLP-1 RA, received approval for treatment of T2D in 2005. Nowadays, various GLP-1 RA drug products are clinically prescribed and used in T2D. These medications also aid in weight loss, reduce the risk of hypoglycemia, and contribute to cardiorenal protective effects along with providing good glycemic control [26]. For the treatment of T2D, Semaglutide (Ozempic), which is also a GLP-1 RA, is widely used. The subcutaneous administration of Semaglutide as an add-on treatment along with Metformin has been approved by the USFDA and EMEA. An oral form of Simaglutide to be administered once a week is also available [27].

## 4. Tirzepatide

In early 2016, Eli Lilly and Company (Indianapolis, IN, USA) first applied a method of glycemic control using tirzepatide [28]. On 14 May 2022, Eli Lilly unlocked one more achievement by receiving US FDA approval for the highly anticipated anti-diabetic drug Mounjaro^®^ (tirzepatide). Tirzepatide is a peptide molecule that is produced synthetically that acts on both GIP and GLP-1 receptors as a receptor agonist. Due to this unique dual activity property, it is also referred to as ‘twincretin’. Subcutaneous administration once a week is adequate, as it possesses a half-life of about 5 days [29].

### 4.1. Structure and Activity

**Figure 2:**
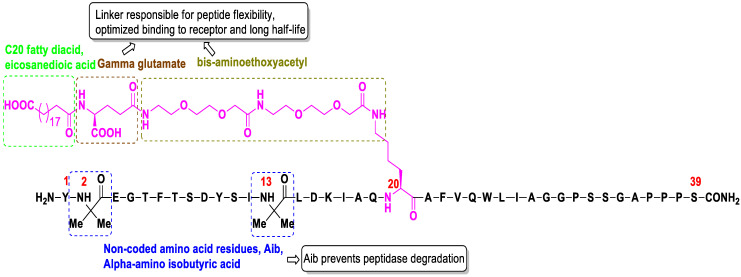
Structural features of tirzepatide, amino acids are denoted as single-letter codes.

Tirzepatide is a synthetic linear peptide molecule containing 39 amino acids. Residues derive from GLP-1, GIP and semaglutide, and a few residues are unique [30]. More specifically, the structure is based on the native GIP sequence and includes C20 fatty diacid moiety (eicosanedioic acid) linked via hydrophilic linkers (γ-Glu-2xAdo, gamma glutamate and bis-aminodiethoxyacetyl) connected to lysine residue at C20 position [31]. The peptide sequence of tirzepatide contains two non-coded amino acid residues (Aib, α-amino isobutyric acid) at position 2 and 13, which are responsible for its long half-life and high affinity to albumin [32]. The C-terminus of the peptide is amidated (Figure 2) [33]. The molecular formula of tirzepatide is C_225_H_348_N_48_O_68_ and the molecular weight is 4813.45. Tirzepatide is the first agent that functions as a dual agonist for the two main human GLP-1 and GIP incretins, a promising drug against both T2D and obesity [34,35,36,37]. It has impressive glycemic efficacy. Moreover, it is the first effective drug to have demonstrated notable body weight loss in a phase 3 study in patients with T2D [38,39,40].

Tirzepatide has significantly better therapeutic efficacy than current drugs [20,41]. It is superior to semaglutide [33,42] and insulin degludec [33,43]. The main critical improvements are modification of residues in the peptide backbone to obtain GIP receptor-activating activity, prolongation of the C-terminus with a sequence of C-terminal of exenatide, and conjugation of the fatty acid side chain to prolong half-life (116.7 h) [30,44]. In addition, hepatoprotection cannot be overlooked [45]. The structural basis of tirzepatide in terms of its functional versatility has been reported [46], and its physiological mechanisms in T2D described [47]. In the human clinical trial (NCT03951753), tirzepatide was compared to semaglutide and placebo with respect to T2D patients’ responses to blood sugar levels after a meal over 28 weeks. It was noted that tirzepatide significantly improved clamp disposition index when compared to both semaglutide and placebo. This in turn showed significant improvements in total insulin secretion rate and insulin sensitivity for tirzepatide. Upon meal tolerance testing, tirzepatide was also noted to slow glucose excursions when compared with placebo. As such, tirzepatide was found to be efficacious in treating T2D [47,48].

### 4.2. Synthesis

**Figure 3:**
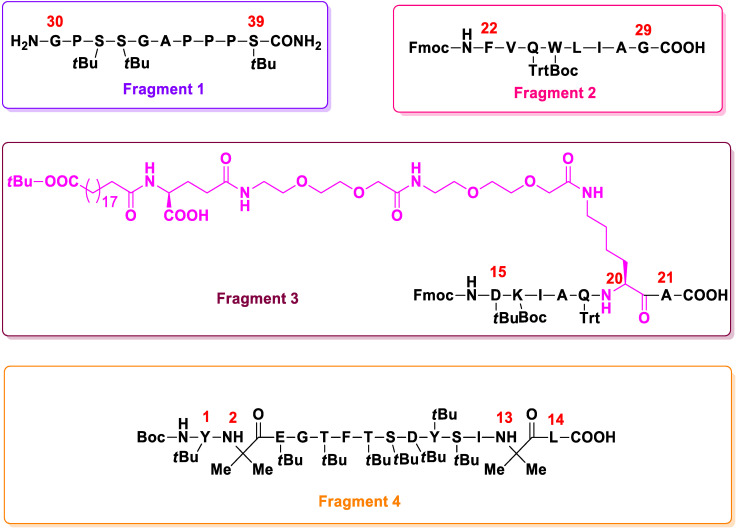
Fragments used to synthesize tirzepatide using the SPPS/LPPS approach.

Recently, Micheal O. Frederick and co-workers from Eli Lilly and company demonstrated a continuous kilogram-scale GMP manufacturing method for the synthesis of tirzepatide using a hybrid solid-phase peptide synthesis/liquid-phase peptide synthesis (SPPS/LPPS) approach [49]. This strategy also includes nanofiltration for purifying the intermediates, and real-time analytical monitoring, which resulted in the development of robust synthetic process with high purity and yield. Considering both the pros and cons of SPPS and LPPS, the researchers selected the four fragments (Figure 3) that had the highest purity and were readily isolable for the synthesis of tirzepatide. These four fragments were synthesized through SPPS, and the coupling of these fragments was carried out using LPPS.

The LPPS portion of the synthesis consisted of four steps and was carried out in a plug-flow reactor (PFR) [50]. In step 1, fragment 1 and 2 were coupled. Separate solutions of fragment 1 and 2 in dimethylsulfoxide (DMSO)/acetonitrile (ACN), [Ethyl cyano(hydroxyimino)acetato-*O*^2^]tri-1-pyrrolidinylphosphonium hexafluorophosphate (PyOxim) in ACN, and *N*,*N*′-diisopropylethylamine (DIPEA) were made previously and brought together in flow prior to entering the PFR. The PFR was coupled with an HPLC for reaction monitoring. Once coupling was performed, the coupled fragment was deprotected using diethylamine (DEA). The deprotected product was then purified using nanofiltration [51], a membrane-based technology, where components of the mixture could be separated based on their molecular weight and hydrophobicity. Similarly, fragment 3 was then coupled with the coupled product of fragments 1 and 2 in step 2, and so on for step 3, in which fragment 4 was coupled. In the final step, 19 acid-labile protecting groups were cleaved using trifluoroacetic acid (TFA). Thus, 8.71 kg pure tirzepatide was isolated, representing an overall yield of 81%. The described hybrid approach decreased the manufacturing risk and also created a robust continuous manufacturing method affording high purity and yield.

### 4.3. Clinical Development

**Figure 4:**
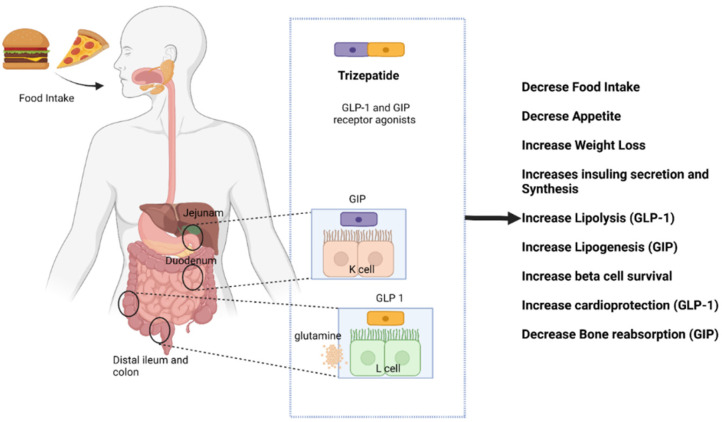
Mechanisms of action of tirzepatide within the human body.

Phase 1 clinical trials, which lasted for 4 weeks, followed by 4 weeks of safety investigation, for tirzepatide showed a remarkable statistical decrease in HbA1c, and postprandial glucose levels were also found to be diminished. A 26-week-long phase 2 clinical trial, which also included a dulaglutide group, showed better efficacy over dulaglutide. It also contributed to reduction of weight and decreased appetite in the subjects. In the SURPASS-1 phase 3 clinical trials, which included six countries worldwide, patients who had ongoing treatment with SGLT2 inhibitors (e.g., dapagliflozin) were included. Some trials included consequent administration of tirzepatide with dapagliflozin, which resulted in higher HbA1c reduction and weight loss [52]. In addition, in the SURPASS-2 phase 3 clinical trial, which compared tirzepatide with metformin to the selective GLP-1 receptor agonist semaglutide, 1 mg administered once a week [53] showed additive effects. Some other additional effects of tirzepatide were noted to lowering of the concentrations of very low-density lipoproteins and triglycerides, in addition to a reduction in blood pressure, as well as an elevation in high-density lipoprotein concentration. The adverse events most commonly reported were nausea, vomiting, and diarrhea, which were mild to moderate and occurred mostly during the dose-escalation period [40,54]. Further, in a phase 2 human clinical study with tirzepatide in conjunction with regulated nutrition and lifestyle with or without metformin, a dose-dependent impact on HbA1c and weight loss was observed, which was larger than that of the selective GLP-1 RA dulaglutide. Meanwhile, in a 12-week phase 2 trial with moderate dose-escalation regimens, gastrointestinal tolerability improved when starting with a lower dose and slowly increasing to the highest effective doses [55]. Mechanism of action of trizepatide is presented in Figure 4.

### 4.4. Pharmacokinetics

In pharmacokinetic (PK) studies in healthy volunteers for doses ranging from 0.25 to 15 mg peak plasma concentration (C_max_), it was found to be dose proportional ranging between 26 and 874 ng/mL. The maximal concentration (T_max_) for tirzepatide was observed after 1–2 days of administration, and the mean half-life (T_1/2_) was found to be 116.7 h (i.e., 5 days), thus favoring a weekly dose regimen [56]. Steady-state exposure with a once-a-week dose was found to be reached after 4 weeks, and the accumulation index was found to be 1.6 during these 4 weeks. In patients with T2D, PK parameters were studied for a final dose of 15 mg, and C_max_ was noted to be 1250 ng/mL, while T_max_ was found to be 24 h [53]. In addition, further PK studies in healthy volunteers over 28 days incorporated a dose escalation up to 10 mg, followed by an oral glucose tolerance test after 23 days of the initial dose, along with a placebo arm of dulaglutide at a dose of 1.5 mg weekly. A significantly improved glucose response was shown compared with placebo for the glucose area under the curve (AUC _0-2h_) on the 23rd day following the oral glucose tolerance test. A reduction in body weight of around −4.05 kg was found in the 10 mg dose subjects [26].

In summary, peptide molecules like semaglutide and tirzepatide have provided promising results for reducing HbA1c and reducing body weight in human phase 1 and phase 2 clinical trials. The SURPASS-1 to SURPASS-5 human clinical trials provided favorable results in comparison with similar moieties like semaglutide and dulaglutide [57,58]. The US FDA approval for tirzepatide is believed to have provided a step forward in the management and treatment of T2D and achieving weight loss, acting as a one-stop solution.

## 5. Conclusions

T2D and obesity are deep-rooted diseases that have no specific cure so far, but can be kept under control by proper application of therapy and treatment, as well as incorporating lifestyle modifications. The alarming increase in the number of patients worldwide requires new scientific developments in order to ease administration, reduce the frequency of dosing, and address multiple issues within a single medication. Tirzepatide has shown promising results in terms of reducing HbA1c and reducing body weight in phase 1 and phase 2 clinical trials. SURPASS-1 to SURPASS-5 human clinical trials have given favorable results by comparison with similar moieties such as, semaglutide and dulaglutide. The USFDA has approved tirzepatide under the brand name Mounjaro, which has become a revolutionary agent for the management and treatment of T2D and achieving weight loss [29,59,60]. Patient compliance and dose adherence are also favored, since it has the advantage of a once-a-week dose administration.

Thus, tirzepatide could be a breakthrough in the treatment of T2D. As such, further research in synthetic peptide therapeutics will gain increasing momentum from now on.

## Data Availability

Not applicable.

## Sample Availability

Samples of the compounds are not available from the authors.
