# Correction: Chavda et al. Tirzepatide, a New Era of Dual-Targeted Treatment for Diabetes and Obesity: A Mini-Review. *Molecules* 2022, *27*, 4315

**DOI:** 10.3390/molecules30061190

**Published:** 2025-03-07

**Authors:** Vivek P. Chavda, Jinal Ajabiya, Divya Teli, Joanna Bojarska, Vasso Apostolopoulos

**Affiliations:** 1 Department of Pharmaceutics and Pharmaceutical Technology, LM College of Pharmacy, Ahmedabad 380009, India; 2 Department of Pharmaceutics Analysis and Quality Assurance, LM College of Pharmacy, Ahmedabad 380009, India; 98jinal@gmail.com; 3 Department of Pharmaceutical Chemistry, LM College of Pharmacy, Ahmedabad 380009, India; divya.teli@lmcp.ac.in; 4 Institute of General and Ecological Chemistry, Faculty of Chemistry, Lodz University of Technology, 116 Żeromskiego Street, 90-924 Lodz, Poland; 5 Institute for Health and Sport, Victoria University, Melbourne, VIC 3030, Australia; 6 Australian Institute for Musculoskeletal Science (AIMSS), Immunology Program, Melbourne, VIC 3030, Australia

## Citation Correction

There was an error in the original paper [1] related to the order of citations. Reference Citations 55 and 56 were intended for the Introduction, not the main text. The authors decided to remove these citations to avoid confusion. The remaining citations were renumbered and ordered in the manuscript.

The authors apologize for any confusion this may have caused. The authors state that the scientific conclusions are unaffected. This correction was approved by the Academic Editor. The original publication has also been updated.
